# Functional exploration of free and encapsulated probiotic bacteria in yogurt and simulated gastrointestinal conditions

**DOI:** 10.1002/fsn3.1254

**Published:** 2019-11-07

**Authors:** Muhammad Afzaal, Azmat Ullah Khan, Farhan Saeed, Aftab Ahmed, Muhammad Haseeb Ahmad, Abid Aslam Maan, Tabussam Tufail, Faqir Muhammad Anjum, Shahzad Hussain

**Affiliations:** ^1^ Institute of Home & Food Sciences Government College University Faisalabad Faisalabad Pakistan; ^2^ Department of Food Science and Human Nutrition University of Veterinary and Animal Sciences Lahore Pakistan; ^3^ National Institute of Science & Technology University of Agriculture Faisalabad Pakistan; ^4^ The University of the Gambia Gambia Gambia; ^5^ College of Food and Agricultural Sciences King Saud, University Riyadh Saudi Arabia

**Keywords:** encapsulation, gastrointestinal conditions, probiotics viability, yogurt

## Abstract

The core objective of the current study was to evaluate the effect of microencapsulation on the viability and stability of probiotic bacteria in yogurt and simulated gastrointestinal conditions. For this purpose, probiotic bacteria were encapsulated with sodium alginate and carrageenan by encapsulator. Yogurt was prepared with the incorporation of free and encapsulated probiotic bacteria and was analyzed for physicochemical, microbiological, and sensorial attributes. Encapsulation and storage exhibited a significant (*p* < .05) effect on different parameters of yogurt. An increasing trend in syneresis and acidity while a decreasing trend in viscosity, pH, viability, and stability were observed. The value of syneresis increased from 2.27 ± 0.17 to 2.9 ± 0.14 and acidity from 0.48 ± 0.04 to 0.64 ± 0.01 during 4 weeks of storage. The value of viscosity decreased from 3.68 ± 0.21 to 2.42 ± 0.09 and pH from 4.88 ± 0.31to 4.43 ± 0.36 during 28 days of storage. Unencapsulated (free) cells exhibited poor survival. The viable cell count of probiotic bacteria in the free‐state in yogurt was 9.97 logs CFU/ml at zero‐day that decreased to 6.12 log CFU/ml after 28 days. However, encapsulation improved the viability of the probiotics in the prepared yogurt and GIT. The cell count of probiotics encapsulated with sodium alginate and carrageenan was 9.91 logs CFU/ml and 9.89 logs CFU/ml, respectively, at zero‐day that decreased to 8.74 logs CFU/ml and 8.39 log CFU/ml, respectively. Free cells (unencapsulated) showed very poor survival. Similarly, during in vitro gastrointestinal assay, the survival rate of encapsulated probiotic bacteria in simulated gastric solution and intestinal solutions was higher than that of free cells. In the case of encapsulated bacteria, only 3 logs while for free cells, 7 log reduction was recorded. Sodium alginate microcapsules exhibited better release profile than carrageenan. Conclusively, microencapsulation improved the survival of probiotic bacteria in carrier food as well as in simulated gastrointestinal condition.

## Graphical abstract

Encapsulation was done by encapsulator using two coating materials and was incorporated in yogurt in free and encapsulated forms. To access the viability, unencapsulated and encapsulated probiotics were subjected to simulated gastrointestinal conditions. The results indicated that encapsulation provide the protection in carrier food and in gastrointestinal conditions.

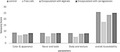

## 1 INTRODUCTION

The functional foods improve human health apart from the native nutritional value. The traditional concept of food has changed and now, consumers demand therapeutic and curative foods. Owing to this, there is a consistent rise in the demand for functional foods supplemented with probiotics. There is a high demand for the manufacturing of modern food products owing to their improved taste and health impacts (Fito et al., 2001; Yağcı & Göğüş, 2008). The awareness of the functional foods has increased their marketing demands throughout the world (Agrawal, 2005). In previous millennia, dairy foods containing probiotic bacteria have been investigated extensively. “Probiotics are the live microorganisms which when administered in adequate amounts confer health benefit on the host” Hill et al., 2014. Various functional dairy foodstuffs are manufactured through fermented milk, beverages, and yogurts. The manufacturing of food with added prebiotics is a successful application as means of probiotic bacteria (Hashemi, Gheisari, and Shekarforoush (2015); Da Silva, Fátima, Olbrich dos Santos, & Pinto Correia, 2015). Probiotics confer certain important health benefits when consumed as food components or supplements (Sanders & Marco, 2010). Probiotics refer to the wide range of microorganisms especially bacteria as well as yeast. Most widely studied group is lactic acid bacteria (LAB), and *Lactobacillus acidophilus* and *bifidobacteria* are considered to be the most important probiotics with beneficial possessions on the human gastrointestinal (GI) pathway (Burgain, Gaiani, Linder, & Scher, 2011). Probiotics are found to be present in the wider range of food products especially in dairy products (Balthazar et al., 2018; Stella et al., 2005).

To draw health benefits from probiotics, these must be present in sufficient amounts in foods, the recommended level of probiotics in the carrier foods ranges from 10^6^ to 10^7^ CFU/ml (Champagne, Ross, Saarela, Hansen, & Charalampopoulos, 2011). Probiotic viability in all type of food products is affected by many intrinsic and extrinsic aspects such as oxygen, postacidification in fermented products, pH, storage temperature, production of hydrogen peroxide, and harsh processing conditions (Shah, 2000). Freezing injury and oxygen toxicity decrease the viability and stability of probiotic bacteria in dairy products (Vasilyevich & Shah, 2008). The endurance of probiotic bacteria in the gastrointestinal situation is very important for health‐promoting properties of the probiotics (Shi, Li, Zhang, et al., 2013).

Various protection methods are existing that can improve the viability of probiotic bacteria; however, still, the survival and success rate is lower. Currently, the encapsulation system is gaining much attention as it improves the endurance of probiotic bacteria in carrier foods as well as in the GIT system (Kanmani et al., 2011). Microencapsulation is the method/technique by which an ingredient (solid, liquid, and gas) is coated with a wall material (Muzzafar & Sharma, 2018). The bioactive ingredient can be a living (probiotic) or nonliving (vitamin, minerals) Encapsulation with different encapsulating materials has successfully shown to protect probiotic bacterial in many fermented dairy products (Abghari et al., 2011). Among the available materials for encapsulation, the sodium alginate and chitosan are widely utilized because these materials are nontoxic, economical, and well easy to handle (Krasaekoopt, Bhandari, & Deeth, 2006). Many researchers have reported that encapsulating materials (chitosan, alginate, xanthan, carrageenan, starch gelatin, and vegetable gum) have a significant protective effect on the endurance of *Bifidobacteria bifidum* and *L.* *acidophilus* within carrier foods and gastrointestinal system. Double‐layered chitosan–alginate beads encourage the endurance of the cell in the substantially acidic products like the juice of pomegranate (Nualkaekul, Lenton, Cook, Khutoryanskiy, & Charalampopoulos, 2012).

*Bifidobacterium* and *Lactobacillus* are considered as important probiotics owing to their beneficial impacts on human health (Saxelin, Tynkkynen, Mattila‐Sandholm, & Vos, 2005). The therapeutic characteristics of some probiotic foods show dependence on the viability of these probiotic bacteria. Significance and selection of carrier foods for probiotics are of great importance. Among the carrier foods, dairy products are highly accepted by consumers and play an important role in enhancing the target delivery (Gill & Prasad, 2008). Fermentation of milk with probiotic bacteria decreases the mineral loss and improves the nutritional status of the dairy products (Nadelman et al., 2017). The starter culture, particularly *Lactobacillus *spp. isolated from the camel milk, can have more benefits in terms of the nutritional profile for the dairy products (Ayyash, Al‐Nuaimi, Al‐Mahadin, & Liu, 2018). Due to the great diversification of dairy products, the milk industry possesses the potential to proportionate an additional benefit to consumers' health (Balthazar et al., 2018).

Yogurt predominantly probiotic yogurts endorse many health benefits by providing natural nutrients. Probiotic yogurt is also enriched with intestinal microbiota that has a positive impact on intestinal microflora. (Cruz et al., 2013). Yogurt among fermented product is a unique and significant source for the induction of probiotics and prebiotics in the human diet. Therefore, there is a great potential for the development of functional foods like probiotic yogurt. Keeping in view the importance of probiotics and yogurt, the present study was designed to probe the effect of encapsulation on the viability of probiotics in food and simulated conditions. The objectives of the present study were to encapsulate the probiotics with cost–effective coating materials and evaluate their survival under different conditions.

## 2 MATERIALS AND METHODS

### 2.1 Probiotic culture activation

A pure culture of probiotic bacteria (*L. acidophilus ATTC‐4356*) was obtained in freeze‐dried form National Institute of Food Science & Technology, University of Agriculture Faisalabad. The activation was done by inoculating the culture in the MRS‐broth at 37°C for 24 hr. The probiotic cells were centrifuged and thereafter, washed in sterile saline solution with the same centrifugation process. The obtained probiotic cells were used in the encapsulation process. The cell concentration was adjusted at more than 10^9^CFU/ml.

### 2.2 Encapsulation of probiotics

*Lactobacillus acidophilus* was encapsulated with sodium alginate and carrageenan microgels by adopting the method of Yeung, (Yeung, Arroyo‐Maya, McClements, & Sela, 2016) with slight modification. Solutions of carrageenan (2.0% w/v) and sodium alginate (2.0% w/v) were prepared and sterilization was done at 121°C (15psi) for 15 min. The germ‐free solutions of polysaccharides were combined with 2 ml of 10^9^ CFU/ml probiotic organisms present in a physiological saline solution. The beads were encapsulated aseptically by using an encapsulator (B‐390, Buchi‐Switzerland) under standard operating conditions. Liquors of encapsulating materials were injected into hardening solutions of 0.1 M calcium chloride solution. After an hour, beads were obtained by the filtration process and washed by sterile deionized water. The beads were stored in physiological saline solution at 4°C for future use.

### 2.3 Characterization of beads

The size or diameter of the encapsulated cells was determined by stage micrometer. For the determination of size and shape, the prepared beads were examined under a light microscope.

### 2.4 Entrapment efficiency

The efficiency of the encapsulation of probiotic bacteria was investigated according to the method followed by Chavarri (Chávarri et al., 2010) with slight modifications. Encapsulated probiotics were disintegrated in phosphate buffer by using stomacher (Seward, UK), and afterward, the amount of entrapped cells was calculated by the pour plate technique. The results were expressed as a number of colonies forming units/bead (CFU/bead).

The value of encapsulated efficiency was evaluated by using the following relationship:

$$\text{Encapsulation Efficiency}=\text{EE}\;=\;\frac{{\text{Log}}_{10}N\times 100}{{\text{Log}}_{10}N_{0}}$$

where, N is the number of viable entrapped cells released from the encapsulated beads. N_0 _is the number of free cells added to the biopolymer mixture prior to encapsulation.

### 2.5 In vitro studies

Survival of free and encapsulated probiotics under simulated conditions.

The viability/survival of free and encapsulated probiotics was assessed by adopting the method of Damodharan (Damodharan, Palaniyandi, Yang, & Suh, 2017) with slight modification. Briefly, the simulated solution of gastric and intestinal was prepared. The simulated gastric juice was prepared by adding 0.03 M phosphate buffer and 10 mg/ml pepsin in test tube containing warmed water. The pH was adjusted to 2 by using I M HCL. The prepared solution was mixed with free and encapsulated probiotics and incubated at 37°C for a described period of interval. The preparation of simulated intestinal juice was carried out as previously described. In short, 0.03 M phosphate buffer, 10 mg/ml trypsin, and 0.3% bile salt were added to sterilized water. pH was adjusted to 7.5 by using 1 M NaOH. The prepared solution was mixed with free and encapsulated probiotics and incubated at 37°C. The survival of encapsulated and free suspending cells was recorded at predetermined time periods (0, 30, 60, 90, and 120 min).

### 2.6 Manufacturing of probiotic yogurt

Yogurt was manufactured by the following method as described by Schoina et al., (2015). Commercial pasteurized and homogenized milk was purchased from the local market. Milk analysis was carried out by following (AOAC 2006) methods. Milk was heated and cooled down to 45°C. The milk was poured in already germ‐free cups (100 ml each) and kept in incubator for fermentation at 42–45°C for 6–8 hr. Each sample was inoculated with 1:1 proportion of yogurt culture that is *Streptococcus thermophiles* and *L. bulgaricus*. The probiotic yogurt was manufactured by adding probiotics to unencapsulated (free) and encapsulated (sodium alginate and carrageenan). 0.1 g (approx10.45 CFU) of microbeads was added to yogurt sample. The following treatments were made T1. Control, T2: Yogurt containing free cells, T3: *Yogurt containing encapsulated L. acidophilus* with alginate, T4: *Yogurt containing encapsulated L. acidophilus* with carrageenan.

### 2.7 Product analysis

Probiotic yogurt was subjected to microbiological, physicochemical, and sensorial analysis. Yogurt was analyzed for pH, lactose, viscosity, viability, and storage stability.

### 2.8 Enumeration of storage stability and viability of free and encapsulated probiotics in yogurt

Enumeration of probiotic bacteria was performed following the protocol used by Pinto et al., (2012). Yogurt was stored at 4°C and was analyzed with an interval of 0, 7, 14, 21, and 28 days. Yogurt samples of all treatments were taken to calculate the viable cells. Yogurt samples of all treatments were diluted to 100 ml with saline solution and mixed with a stomacher and spread on MRS medium, incubated at 37°C for 48 hr. The viable count was calculated. Experiments were repeated for three times (AOAC, 2006).

### 2.9 pH

It was determined by using a digital pH meter. pH meter was calibrated by using a buffer solution (Granato, Araújo Calado, & Jarvis, 2014).

### 2.10 Viscosity

Viscosity was determined by using viscometer (Brookfield LVDVE‐230) by adopting a method of Amerinasab, Labbafi, Mousavi, & Khodaiyan, (2015). All readings were recorded in centipoise (cp) unit.

### 2.11 Syneresis

Syneresis was determined by the method described by Ayar & Gurlin, (2014).

### 2.12 Sensory analysis

Sensory evaluation of yogurt was carried out by adopting the protocol described by Sheu & Marshall, (1993). Sensory evaluation was done by expert panellists of Department of Food Sciences. All of the judges were nonsmokers and trained for evaluating dairy products by using a 9‐point hedonic scale for taste, flavor, color, body, texture, appearance, and overall acceptance.

### 2.13 Statistical analysis

The data obtained for each parameter were subjected to analysis of variance (ANOVA). Data were compared by a two‐way analysis of variance. All results are expressed as mean ± *SD*. Differences were considered significant at *p*\.05. Four replicates of each treatment were analyzed with predefined intervals.

## 3 RESULTS AND DISCUSSION

### 3.1 Encapsulation entrapment efficiency

It has been reported that coating with wall materials improves the survival and stability of probiotics in processing and in simulated gastrointestinal conditions (Iyer & Kailasapathy 2005). The effectiveness and survival of probiotics depend on the type of probiotic and the used wall materials. A variation in encapsulation yield was observed in sodium alginate and carrageenan as a coating material (Table 1). Statistically, the yield of the *L. acidophilus* cells encapsulated with sodium alginate was higher than those of encapsulated with carrageenan. In Table 1, a comparison of the encapsulation efficiency between two coatings can be observed that showed a significant difference (*p* < .05).

**Table 1:** Encapsulation efficiency

Type of matrix	Numbers before encapsulation	Numbers after encapsulation	Efficiency (%)
Sodium Alginate	8.68 ± 0.06	8.47 ± 0.04	98%
Carrageenan	8.44 ± 0.04	8.13 ± 0.05	96%

### 3.2 Mean diameter of the encapsulated beads

The mean values for the diameter of the encapsulated beads were recorded, and the data showed that beads prepared with sodium alginate had a mean value of 714 µm while for carrageenan beads had mean diameter of 726 µm. The obtained results clearly showed that the diameter of beads is affected by the type of coating materials. A similar finding was found earlier by Shi et al., (2013). Encapsulating materials, as well as the diameter of the capsules, affect the viability of the probiotics. Undue reduction in diameter can remove the protective function of encapsulation, whereas, increasing capsule diameter decreases digestibility by pancreatic enzymes.

### 3.3 Stability of the encapsulated probiotic bacteria in simulated gastric conditions

**Figure 1:**
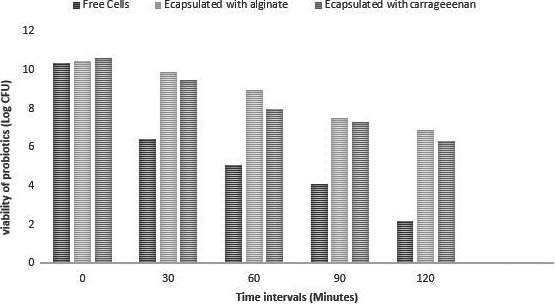
Survival of free and encapsulated probiotics under simulated gastric conditions. Probiotic survival (Log _10_ CFU/ml) of free (unencapsulated) and encapsulated with (sodium alginate and carrageenan) in simulated gastric conditions in time interval (0, 30, 60, 90, 120 min)

Cell viability in the stomach and intestinal conditions is important in order to get the desired benefits of probiotics. Free and encapsulated cells were exposed to activated gastric juice. An instant decrease was examined in free cells in contrast to the cells encapsulated with sodium alginate and carrageenan (Figure 1 **)**. The sodium alginate capsules showed 3.57 log reductions while carrageenan capsules showed 4.28 log reduction as compared to unencapsulated cells that is 8.18 log. The encapsulation of the cells within either sodium alginate or carrageenan significantly affected (*p* < .05) on the survival of probiotic bacteria. The results demonstrated that encapsulation provides protection to probiotics in simulated gastric conditions. The results are also in accordance with Qi, Liang, Yun, & Guo, (2019) who found 60% increased survival rate of probiotics under simulated gastrointestinal conditions compared to free cells (25%) and concluded that the encapsulation by using biopolymers offers an effective way to protect probiotics in adverse processing and in vitro conditions.

### 3.4 Stability of the encapsulated probiotic in intestinal conditions (pH 7.5)

**Figure 2:**
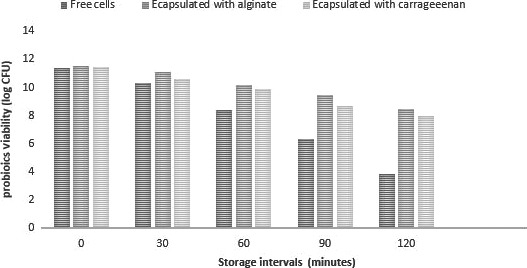
Survival of free and encapsulated probiotics under simulated intestinal conditions. Probiotic viability (Log _10_ CFU/ml) of free (unencapsulated) and encapsulated with (sodium alginate and carrageenan) in simulated intestinal conditions in time interval (0, 30, 60, 90, 120 min)

Different encapsulating materials showed a protective effect on probiotics when exposed to intestinal conditions. In the current study, free and encapsulated cells were exposed to the simulated intestinal artificial solution for a specific interval of time. A rapid reduction in unencapsulated cells was observed as compared to the encapsulated probiotics at pH 7.5 (Figure 2). The encapsulation of the cells with either sodium alginate or carrageenan had a statistically significant effect (*p* < .05) on cell survival. The sodium alginate and carrageenan capsules showed slow log reduction as compared to free cells. The present findings are also in accordance with Shah and Ravula, (2000), who reported that alginate coating improves the release and survival in GIT conditions. The results demonstrate that encapsulation with biopolymers is a useful tool for the longevity of sensitive ingredients. The results are also in accordance with Qi et al., (2019) who found 60% increased survival rate of probiotics under simulated gastrointestinal conditions compared to free cells (25%) and concluded that the encapsulation by using biopolymers offers an effective way to protect probiotics in adverse processing and in vitro conditions.

### 3.5 Product analysis

Prepared yogurt was subjected to various physicochemical and microbiological analyses to assess the effect of coating materials on different attributes.

### 3.6 The viability of free (unencapsulated) and encapsulated probiotic bacteria in yogurt

**Figure 3:**
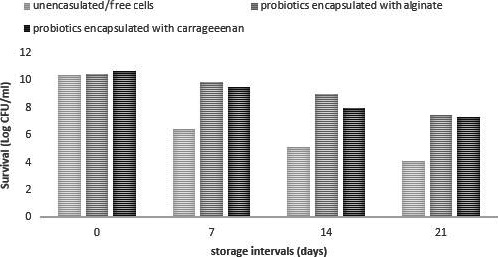
Viability of free and encapsulated probiotics in yogurt. Viability (Log 10 CFU/ml) of free (unencapsulated) and encapsulated (with Sodium alginate and carrageenan) probiotic bacteria (*Lactobacillus acidophilus*) in yogurt during storage intervals (0, 7, 14, 21 days). Each bar represents mean value for the viability of probiotics

The probiotic bacteria were encapsulated with alginate and carrageenan and incorporated into yogurt to evaluate their stability in this dairy product. The added probiotics were enumerated from day one and continued with an interval of 7 days until 28 days of storage (Figure 3). Free/unencapsulated probiotic bacteria showed a rapid drop in log reduction from day one to 28 days of storage at −20°C; whereas, probiotic bacteria encapsulated in alginate as well as carrageenan showed slow log reduction. The obtained results demonstrated that the numbers of viable probiotic bacterial cells decreased during storage. Further, it has been observed that the death rate of encapsulated cells was statistically lower as compared to free cells in yogurt. Similar results were found by Homayouni, Azizi, Ehsani, Yarmand, & Razavi, (2008) that encapsulation improves the survival of probiotics in the frozen dairy products. The results are also in accordance with Qi et al., (2019) who found 60% increased survival rate of probiotics under simulated gastrointestinal conditions compared to free cells (25%) and concluded that the encapsulation by using biopolymers offers an effective way to protect probiotics in adverse processing and in vitro conditions. These results are also in accordance with findings of Iqbal et al. (2018) who reported survival of probiotics in yogurt. According to Andleeb and Vasudha (Muzzafar & Sharma, 2018) who used different coating materials, used and reported a better survival of encapsulated probiotics in carrier food as compared to unencapsulated cells. Another study reported by Qi et al., (2019) showed the better survival of encapsulated probiotics in yogurt. The number of viable cells in case of encapsulation was decreased by just 1.76 log CFU/g while in case of free were decreased by 4.82 log CFU/g. In a study conducted by Pradeep Prasanna & Charalampopoulos, (2019), the results revealed that significant loss (3.67 log CFU/g) in the survival of unencapsulated probiotics was recorded as compared to encapsulated cells over a period of 28 days.

### 3.7 Effect of free and encapsulated probiotic bacteria on the pH of the yogurt

**Figure 4:**
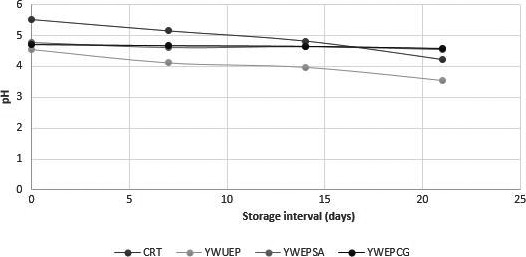
Effect of Free & encapsulated probiotics on pH of yogurt. Effect of free (unencapsulated) and encapsulated (with sodium alginate and carrageenan) on pH of yogurt during storage intervals (0, 7, 14, 21 days) compared with control. Each bar represents mean value for pH of treatments. *CRT‐Control; YWUEP‐yogurt with unencapsulated probiotics; YWEPSA‐yogurt with encapsulated probiotics Sodium alginate YWEPCG‐yogurt with encapsulated probiotic carrageenan*

The pH of probiotic carrier food has a direct relationship with the survival of the probiotic bacteria. The low pH causes a decrease in the survival rate of the probiotics. A decline in pH was observed during storage in all types of yogurt over a period of 28 days of storage (Figure 4). Encapsulated probiotic bacteria cause slower postacidification as compared to unencapsulated cells (Muzzafar & Sharma, 2018).

### 3.8 Effect of free and encapsulated probiotic bacteria on the viscosity of yogurt

A decreasing trend in viscosity of all types of yogurt was observed. The decrease in viscosity could be due to an increase in syneresis during storage. A significant difference in viscosity was observed in all treatments. The highest viscosity was observed in treatment containing carrageenan beads. This could be due to the thickening and stabilizing properties of the biopolymers.

### 3.9 Effect of free and encapsulated probiotic bacteria on the syneresis of yogurt

**Figure 5:**
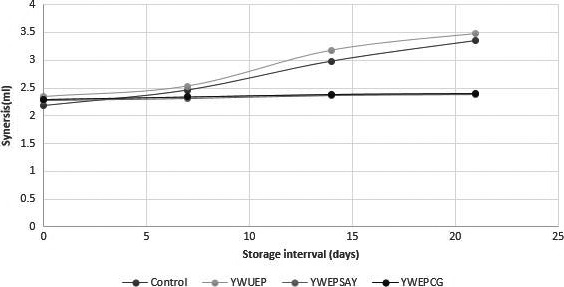
Effect of free and encapsulated probiotics on synersis of yogurt. Effect of free (unencapsulated) and encapsulated (with sodium alginate and carrageenan) on synersis of yogurt during storage intervals (0, 7, 14, 21 days) compared with control. Each bar represents mean value for viscosity of treatments. CRT‐Control; YWUEP‐yogurt with unencapsulated probiotics; YWEPSA‐Yogurt with encapsulated probiotics Sodium alginate YWEPCG‐Yogurt with encapsulated probiotic carrageenan

An increasing trend in syneresis of all types of yogurt was observed. The increase in syneresis could be due to the conversion of solid contents of yogurt into different metabolites by probiotic bacteria during storage. A significant difference in syneresis was observed in all treatments (Figure 5). The highest syneresis value was observed in treatment containing free or unencapsulated probiotics.

### 3.10 Sensory analysis

**Figure 6:**
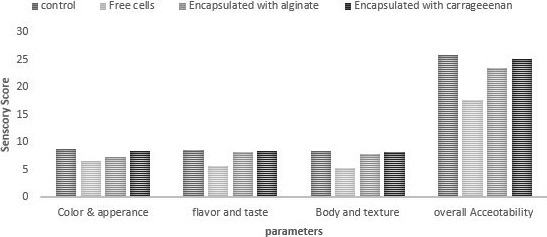
Effect of free and encapsulated probiotics on sensory attributes of yogurt. Effect of free (unencapsulated) and encapsulated (with sodium alginate and carrageenan) on sensory attributes (color, appearance, flavor, taste, body, texture and overall acceptability) of yogurt during storage intervals compared with control. CRT‐Control; YWUEP‐yogurt with unencapsulated probiotics; YWEPSA‐Yogurt with encapsulated probiotics Sodium alginate; YWEPCG‐yogurt with encapsulated probiotic carrageenan

Sensorial characteristics of the products critically affect the acceptance of the newly developed product, and it has become a big challenge for the food industry. The overall acceptability of the prepared yogurt in terms of color, texture, and taste of free and encapsulated probiotic yogurt was noted (Figure 6). From the sensory analysis, it was concluded that the incorporation of free and encapsulated probiotics had significantly affected the sensory properties of yogurt.

## 4 CONCLUSIONS

The findings of the recent study show that encapsulation with both type of polymers has a significant effect on the probiotic viability under simulated gastrointestinal conditions and in carrier food (yogurt). A milder postacidification in case of yogurt containing encapsulated probiotics was observed. Encapsulation had significantly affected the physicochemical and sensory attributes of the yogurt. The results revealed that encapsulation with sodium alginate and carrageenan could be used to enhance the survival of probiotics. Microencapsulation is useful technology to ensure the recommended therapeutic level (10^6^–10^8^ CFU/g) of probiotics in carrier food. Microencapsulation must be adopted by the food industry to ensure the viability of probiotics in functional foods.

## 5 RECOMMENDATIONS

The microencapsulation using sodium alginate and carrageenan must be adopted by food industry for large‐scale production of microbeads for incorporation of functional foods.

## CONFLICT OF INTEREST

Authors declare that they have no conflict of interest.

## ETHICAL APPROVAL

This article does not contain any studies with human participants or animals performed by any of the authors.
